# Rehabilitative interventions in patients with persistent post COVID-19 symptoms—a review of recent advances and future perspectives

**DOI:** 10.1007/s00406-023-01631-9

**Published:** 2023-06-16

**Authors:** Rainer Gloeckl, Daniela Leitl, Tessa Schneeberger, Inga Jarosch, Andreas Rembert Koczulla

**Affiliations:** 1 Institute for Pulmonary Rehabilitation Research, Schoen Klinik Berchtesgadener Land, Malterhoeh 1, 83471 Schoenau Am Koenigssee, Germany; 2 Department of Pulmonary Rehabilitation, Member of the German Center for Lung Research (DZL), University Medical Center Giessen and Marburg, Philipps-University Marburg (UGMLC), Marburg, Germany; 3 Teaching Hospital, Paracelsus Medical University Salzburg, Salzburg, Austria

**Keywords:** Long-COVID, SARS-CoV-2, Rehabilitation, Therapy, Treatment

## Abstract

The SARS-CoV-2 pandemic has not only caused millions of deaths but left also millions of people with persistent symptoms behind. These long-term COVID-19 sequelae cause a considerable burden on individuals´ health, healthcare systems, and economies worldwide given the high rate of SARS-CoV-2 infections. Therefore, rehabilitative interventions and strategies are needed to counteract the post COVID-19 sequelae. The importance of rehabilitation for patients with persistent COVID-19 symptoms has been recently also highlighted in a Call for Action by the World Health Organisation. Based on previously published research, but also in line with clinical experience, COVID-19 is not *one specific* disease but rather presents in different phenotypes that vary in their pathophysiological mechanisms, symptomatic manifestations, and potential interventional approaches. This review provides a proposal for differentiating post COVID-19 patients in non-organ-specific phenotypes that may help clinicians to evaluate patients and to plan therapeutic options. Furthermore, we present current unmet needs and suggest a potential pathway for a specific rehabilitation approach in people with persistent post-COVID symptoms.

## Post-COVID–the burden of the disease

The total number of SARS-CoV-2 infections is still increasing and exceeded the 700 million cases threshold (as registered by the world health organization (WHO) in March 2023) [1]. In terms of the cumulative number of deaths due to COVID-19, there was a significant excess mortality rate that ranged between 2.64 million cases (in high-income countries) up to 5.27 million deaths in (low-income countries) within the first two years of the pandemic [2]. Despite these dramatic high mortality rates, counting only deaths ignores the disability that comes along with persistent post-COVID symptoms. Several studies have assumed that healthy years (health-adjusted life years; QALYs) lost per COVID-19 case ranged from 0.92 (male in his 30 s) to 5.71 (girl under 10) and were 3.5 and 3.6 for the oldest females and males [3, 4].

The WHO named these SARS-CoV-2 sequelae as *post COVID-19 condition* and defined it as “the continuation or development of new symptoms 3 months after the initial SARS-CoV-2 infection, with these symptoms lasting for at least 2 months with no other explanation” [5]. A post COVID-19 condition can potentially affect anyone after being exposed to SARS-CoV-2. However, there is evidence that patients with a more severe acute phase of the disease (including hospitalization) [6, 7], as well as older age and female gender [8] were associated with a greater risk of developing long-term health consequences. The real prevalence of the post COVID-19 condition is hard to evaluate because it is closely related to the method of assessment. So far, studies have assessed different patient cohorts (hospitalized, non-hospitalized, elderly, etc.), asked at various time intervals (i.e. 1, 2, 3, 6 months after infection), and used different survey tools (interview, self-reported, app-based, etc.) [9]. This heterogeneity of assessment methods resulted in an extremely wide range of post-COVID prevalence between 2.3 and 91% [9]. However, also the virus variant may play a role in the development of post COVID-19 sequelae. First evidence suggests that the relative risk of developing post COVID-19 condition in Omicron variant infected subjects is 25 to 50% less than in Delta variant infected people [10].

How many of the symptoms currently attributed to a post COVID-19 condition actually represent an unmasking or an exacerbation of underlying comorbidities or are unrelated to COVID-19 is uncertain. Symptoms that were present before the SARS-CoV-2 infection are often not recorded or only assessed by recall. Findings from a longitudinal population-based study compared symptoms in a sample of 4231 people three to five months after SARS-CoV-2 infection versus 8462 matched controls without SARS-CoV-2 infection [11]. A strength of this study was, that subjects with post COVID-19 condition served as their own control, because the pattern and severity of symptoms were also assessed before SARS-CoV-2 infection. This study found, that in 12.7% (one out of eight patients) infected with SARS-CoV-2, symptoms could be attributed to COVID-19 [11]. A more conservative estimate suggests a prevalence rate of post COVID-19 condition (symptoms for ≥ 12 weeks) to be around 2 to 5% [7]. Even by applying this rather conservative assumption, this means that about 14 to 35 million people worldwide suffer from SARS-CoV-2 sequelae.

An impaired health condition is only one side of the coin–a labor force survey found that 44% of people with post COVID-19 condition were unable to work and 51% worked fewer hours which was directly resulting in lower income [12].

In short, post COVID-19 condition causes a considerable burden on people’s health, health-care systems, and economies worldwide. Given the high rates of SARS-CoV-2 infections, rehabilitative interventions and strategies are needed to counteract the burden of post COVID-19 conditions. The importance of rehabilitation for people with post COVID-19 condition has been recently highlighted in a Call for Action by the WHO [13], which focuses on the adoption of integrated care models to manage the specific needs. In this call, two out of four recommendations refer to rehabilitation, highlighting the need to strengthen health-care systems to provide individualized, multidisciplinary rehabilitation in a systematic and evidence-based manner.

**Fig. 1:**
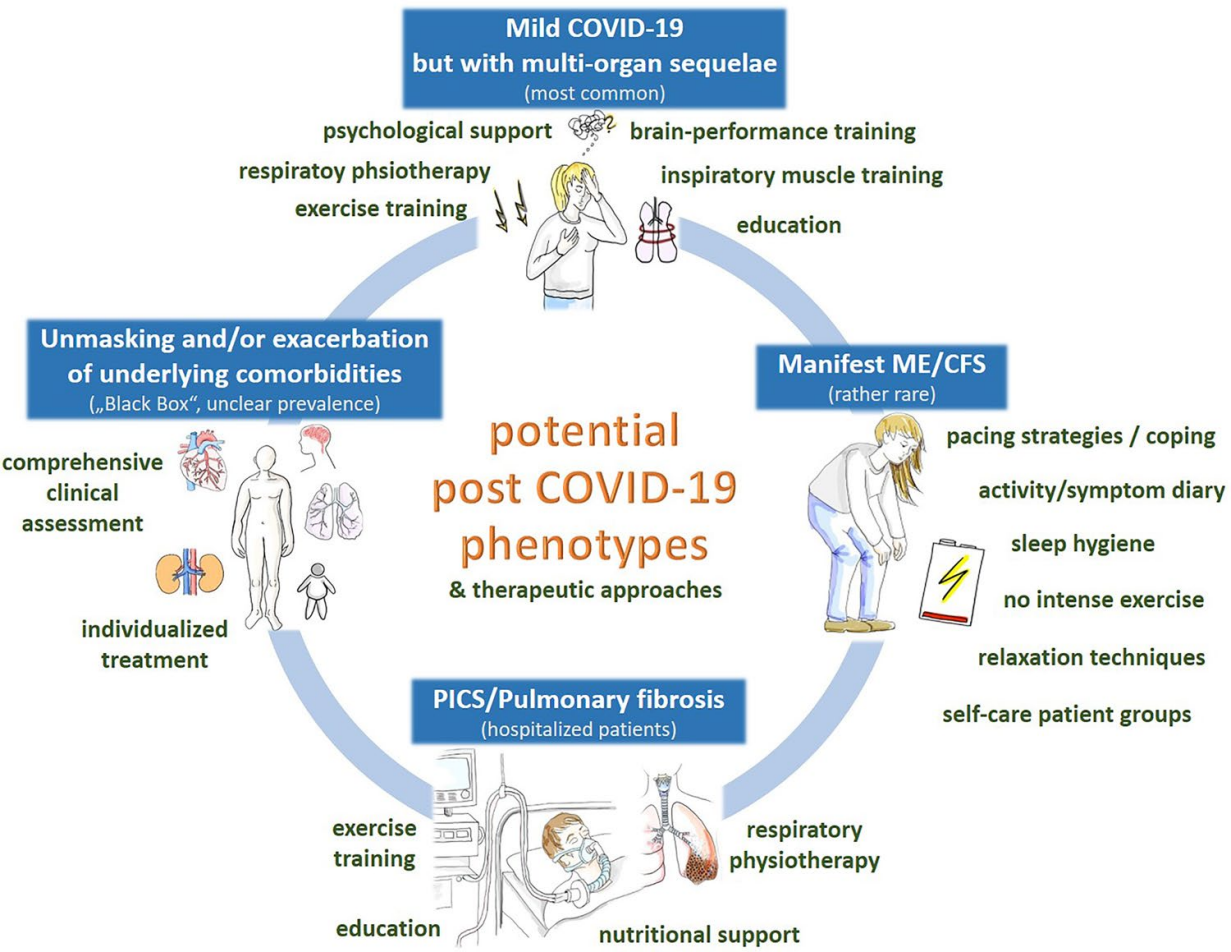
Proposal for non-organ-specific post COVID-19 phenotypes and potential non-pharmacological rehabilitative interventions (in green). *PICS*: Post-intensive care syndrome, *ME/CFS*: myalgic encephalomyelitis/chronic fatigue syndrome

Based on previously published research, but also in line with clinical experience, a post COVID-19 condition is not *one specific* disease but rather presents in different phenotypes that vary in their pathophysiological mechanisms, symptomatic manifestations, and potential interventional approaches [14]. This review provides a proposal for differentiating post COVID-19 patients in non-organ-specific phenotypes (see Fig. 1). This may be helpful for clinicians and may also guide future research to differentiate patients and evaluate therapeutic options for rehabilitation programs.

## Phenotyping patients with post COVID-19 sequelae

More than 200 different symptoms in 10 organ systems have been identified as potential post COVID-19 sequelae [15]. Several studies have investigated the prevalence of the most common post COVID-19 symptoms. A systematic review (including data from 37 studies and 9677 patients) analyzed the prevalence of symptoms, 3 to 6 months after SARS-CoV-2 infection [16]. The most prevalent symptoms were fatigue/weakness (11–42% of patients), dyspnea (8–37%), painful muscles (7–24%), and loss of smell/taste (3–14%). In general, it appears, that neuropsychiatric and pulmonary symptoms are predominant.

## Post-COVID phenotype “*mild COVID-19 but with multi-organ sequelae*”

### Description

At the beginning of the pandemic, COVID-19 was first recognized as a respiratory disease, because many patients developed pneumonia. But COVID-19, even after a mild disease, can also induce extra-pulmonary complications in other organ systems like the cardiovascular, nervous, gastrointestinal, musculoskeletal, or endocrinological systems [17, 18]. Several studies have concluded, that in patients with mild COVID-19 but multi-organ sequelae, fatigue, dyspnea, and cognitive impairments seem to be the most prevalent manifestations [15, 19]. Fatigue, as an overall feeling of tiredness or exhaustion, is a very unspecific symptom that can appear in association with post-viral fatigue syndrome [20]. It is known, that also other virus infections (such as Epstein Bar virus, Q-fever, and the ross river virus) can result in persistent fatigue that may persist for up to 6 months in 12% of subjects [21].

Also, exercise intolerance and muscle weakness are key impairments in many post-COVID patients for multifactorial reasons. It is assumed that a mix of systemic inflammation, viral infection, and persistent physical inactivity/muscle disuse may contribute to decreased neuronal activation, muscle fiber atrophy, as well as alterations in blood flow, and metabolic muscle function [22]. Underlying reasons for exertional dyspnea might be due to impaired lung function, the prevalence of dysfunctional breathing (i.e. hyperventilation syndrome) [23, 24], or a diaphragm muscle weakness [25]. One study also found brain structural abnormalities that were related to COVID-19, which correlated with reduced cognitive function (like impaired memory, verbal fluency, attention, concentration, etc.) [26].

Admittedly, this phenotype presents quite heterogeneous and makes a comprehensive, multidisciplinary assessment mandatory to differentiate underlying pathophysiologic mechanisms and to identify potentially treatable traits for rehabilitation. Potential therapeutic interventions for the most common symptoms fatigue, dyspnea, and cognitive impairment are discussed below.

### Potential rehabilitative interventions

#### For general fatigue symptoms (excluding ME/CFS)

First, it´s important to differentiate general fatigue symptoms from the diagnosis of Myalgic encephalomyelitis or chronic fatigue syndrome (ME/CFS). Therefore, a comprehensive diagnosis consisting of anamnesis, screening questions, physical examination, and laboratory tests is necessary to confirm or exclude ME/CFS [27]. Patients with signs of general fatigue symptoms should have access to education and skills training on energy conservation techniques (Pacing). In addition, these patients (without a manifest diagnosis of ME/CFS) should be introduced to an individualized exercise training program [28].

Also, signs of anxiety disorders and depressive symptoms can develop during a post COVID-19 condition. Therefore, it is necessary, to screen for possible mental disorders and, if necessary, to apply psychological treatments like interpersonal therapy, behavior activation, and problem-solving counseling [28]. In a randomized controlled trial [29], post COVID-19 patients with post-traumatic stress disorder were assigned to a psychological treatment with an additional narrative exposure therapy, or a control group with only psychological treatment. The intervention group showed larger (although non-significant) improvements in anxiety, depression, and sleep quality compared to the control group. However, this lack of significance might be related to the fact that also the control group received an effective therapeutic intervention.

#### For respiratory symptoms

Respiratory physiotherapy is part of pulmonary rehabilitation programs and is well-established in patients with chronic respiratory diseases. Respiratory physiotherapy can be beneficial to reduce resting and exertional dyspnea and improving thoracic mobility, as well as gas exchange [30, 31]. To successfully manage dyspnea and dysfunctional breathing, it is important to provide a combination of education and skill training on self-management strategies (i.e. breathing techniques such as pursed lips breathing, diaphragmatic breathing, huffing, etc.) [28, 30]. In the case of severe dyspnea, also psychological support in stress management can reduce anxiety and as a consequence also dyspnea [28]. In patients with respiratory muscle weakness, the use of inspiratory muscle training (IMT) is an effective intervention to significantly improve respiratory muscle strength, general physical functioning, as well as breathlessness and chest symptoms [32, 33].

In general, several studies have shown, that post COVID-19 patients with respiratory sequelae can achieve improvements in dyspnea, lung function, and exercise performance after pulmonary rehabilitation (even if patients were referred to pulmonary rehabilitation only 6 months after the SARS-CoV-2 infection) [34, 35].

#### For cognitive symptoms

Brain-performance training like computerized training, cognitive/behavioral feedback, and task-specific training with a focus on basic tasks and activities aim to restore cognitive impairments. The focus should be on developing strategies to overcome daily life challenges and improve attention, memory, and executive functions [28]. Potential exercises can consist of memory exercises or strategic games. In addition, developing compensation strategies (like making lists and setting reminders) can contribute to coping better with everyday life challenges [36]. Already a 4-week cognitive-motor training (twice weekly) can be beneficial and can significantly improve cognitive functions of attention and calculation, recall, lingual and action performance [37].

In patients that developed a post-infectious olfactory dysfunction, the application of repetitive deliberate sniffing training of various odors may be considered [38].

## Post-COVID phenotype “*Myalgic encephalomyelitis or chronic fatigue syndrome (ME/CFS)*”

### Description

Myalgic Encephalomyelitis or Chronic Fatigue Syndrome (ME/CFS) is a complex and severely debilitating disease that is often triggered by various viral infections. This condition is known for decades, but it gained increasing attention since also SARS-CoV-2 is suspected to induce ME/CFS. However, the exact underlying mechanisms of the ME/CFS pathogenesis remain still poorly understood [39]. ME/CFS patients usually suffer from excessive fatigue, post-exertional malaise/post-exertional symptom exacerbation (PEM/PESE), pain disorders, sleep disturbances, cognitive impairment (“brain fog”), and neuroendocrine/immune alterations yielding to major limitations in the quality of life [40–42]. Also orthostatic intolerance and autonomic dysfunction are associated with ME/CFS [43]. Orthostatic intolerance may include postural orthostatic tachycardia syndrome (POTS) [27]. Symptoms occur or worsen only on standing or sitting upright from a reclining position for some time and improve by lying down [27, 43]. The pathogenesis of post-COVID POTS is unclear but includes hypotheses on autoimmunity issues, autonomic dysfunction, direct toxic injury by SARS-CoV-2 to the autonomic nervous system, and an invasion of SARS-CoV-2 in the central nervous system [41].

PEM/PESE is defined as the worsening of symptoms that can follow minimal cognitive, physical, emotional, or social activity and worsen 12 to 72 h after the activity and can last for days or even weeks [28]. A brief questionnaire (DSQ-PEM) can be used for example to assess PEM/PESE [44].

Diagnosing ME/CFS is difficult, because the disease symptoms are broad, and may overlap with other chronic conditions. According to the NICE Guidelines [43] the diagnosis of ME/CFS can only be confirmed when the symptoms are present for at least 3 months. Therefore, ME/CFS is a diagnosis of exclusion that can be guided by using established consensus criteria (i.e. Canadian, Fukuda, Oxford, etc.) [39]. Signs of general fatigue are also common symptoms associated with various health conditions. Therefore, it is important to rule out potential reasons for fatigue due to underlying comorbidities or emotional well-being [27]. In addition to a general health assessment, further specific tests like blood examination, disability screening, muscle power testing, and questionnaires can be carried out depending on the clinical presentations [27]. Currently, there are no specific (non-) pharmacological treatments available, that can cure ME/CFS [43].

### Potential rehabilitative interventions

Pacing is an important key strategy in the management of ME/CFS, along with symptomatic medication, as well as the avoidance of exhaustion and mental stress. Pacing strategies aim to learn to control the symptoms and the disease by developing the patient´s own experiences and raising their awareness of what activities are possible without evolving a “crash” (= symptom exacerbation). Furthermore, it´s crucial to recognize and learn, when to interrupt activities and take a preventive break to avoid worsening of symptoms. Keeping a diary about personal activities and developed symptoms can also help to identify possible triggers and activity thresholds [27, 28, 43].

A recent prospective study in patients with post Covid-19 condition and PEM/PESE has investigated the benefits of using a structured pacing protocol over 6 weeks. Patients were able to significantly reduce the mean number of PEM/PESE episodes per week from 3.4 to 1.1 while increasing their activity level. As a consequence, the patients´ quality of life improved also significantly [45].

As a general recommendation, it is advised to keep the individual activity level roughly at 2/3 of the duration and intensity that has previously worsened symptoms [27, 28, 43]. In addition, to prevent further deterioration of physical functioning in ME/CFS patients, it is recommended to include low-intensity activities in the disease management plan like joint mobilization, muscle stretching, balance exercises, or body awareness therapy [43].

There is also some evidence that suggests that cognitive behavior therapy may be a helpful intervention in the management of ME/CFS. Weekly therapy over a period of 8 weeks in patients with ME/CFS has already shown significant improvements in physical and mental fatigue [46]. However, cognitive behavior therapy should be used with considerable care to avoid distress [47].

Potentially useful interventions to counteract pain symptoms could be physiotherapy, relaxation techniques, or meditation. Also, sleep hygiene and a nutritional plan are recommended interventions in the management of ME/CFS patients [27, 43]. Further, it may be useful to improve coping with ME/CFS by getting support via psychotherapy or self-care patient support groups [27, 28, 43].

In the treatment of orthostatic dysfunction and POTS, in addition to drug treatments possible non-pharmacological interventions are recommended. Patients should avoid situations like a warm environment, large food portions, and sudden changes of posture into an upright or standing position [28]. Also, the intake of at least 2 L of water per day including sufficient salt was suggested [27]. Exercise training in an upright position should be avoided as orthostatic intolerance favorable occurs in this position [28]. Other supportive procedures like wearing compression stockings and or sleeping with feet in elevated positions can help to improve symptoms during daily life [27, 28].

## Post-COVID phenotype “*severe to critical COVID-19 with post-intensive care syndrome (PICS)*”

### Description

A subgroup of patients that need treatment in an intensive care unit (ICU) may develop respiratory failure or even acute respiratory distress syndrome (ARDS) including the need for mechanical ventilation. Because of the advances in medicine in the last decade, more patients survive this kind of critical illness. However, survivors of a critical illness often have to deal with significant physical, mental, and emotional symptoms that may yield in a major impaired quality of life which may persist for months and years after hospital discharge. These consequential issues that may affect up to every second ICU survivor and are summarized under the term *post-intensive care syndrome* (PICS). During the acute phase microvascular ischemia, immobility, and metabolic changes can occur. PICS is multifactorial and may be attributed to these points [36] [36]. Pulmonary fibrosis is one of the more severe respiratory sequelae that both, post-COVID and non-COVID, ARDS survivors may develop [48]. The severity of COVID-19 (with ICU stay, invasive/non-invasive mechanical ventilation, longer hospitalization period, and steroid, antibiotic, or immunoglobulin treatments) is associated with the risk of developing a pulmonary fibrosis sequelae [49]. Subpleural reticulation and ground-glass opacities following critical COVID-19 are still prevalent in 34% of patients 1 year later [50]. Typical symptoms of pulmonary fibrosis are severe (exertional) dyspnea, dry cough, chest pain, and impaired exercise performance [49].

Although PICS is not a new condition, a recent study found, that 75% of ICU-treated COVID-19 patients met PICS criteria 3 months after hospital discharge [51]. The prevalence rate of PICS in the pre-pandemic era was assumed to be between 8 to 49% [52]. This increased rate in COVID-19 patients might be related to the longer dependence on mechanical ventilation compared with non-COVID-19 ARDS [53]. Although this comparison must be interpreted with caution, it could be assumed that critically ill COVID-19 patients are at an even higher risk of developing a PICS. Keeping in mind, that there is an increased number of COVID-19 patients that need ICU treatment, there seems also to be an increasing demand for post-ICU care.

### Potential rehabilitative interventions

Proper management of patients with PICS (with chronic inflammation, thrombosis, and/or pulmonary fibrosis) includes appropriate drug treatment. Even the use of antifibrinolytic medication seems to be beneficial in patients with pulmonary fibrosis due to COVID-19 pneumonia [54].

But patients with PICS may also benefit from rehabilitation programs [36]. A recent review stated, that in addition to exercise training after hospital discharge, also early mobilization and mobility training in ICU, respiratory physiotherapy, and nutritional support may contribute to a better recovery process [55]. In general, it is widely accepted, that patients with interstitial lung diseases can benefit from pulmonary rehabilitation to improve exercise capacity, dyspnea, and quality of life [56]. A recent study compared an early pulmonary rehabilitation program in post-COVID-19 patients and non-COVID-19 patients with respiratory failure [57]. Although post-COVID-19 patients had a longer stay in the ICU and a longer mechanical ventilation duration, post-COVID-19 patients achieved a greater increase in the 6 min walk distance (+ 205 m) compared to respiratory failure patients (+ 93 m). This large and rapid recovery in post-COVID-19 patients suggests the effectiveness of an early PR [57]. Another important finding was, that the earlier the rehabilitation program was introduced after ICU discharge and the longer the rehabilitation duration was, the better patients recovered their physical capacity. Another observational study investigated a comprehensive 3-week inpatient pulmonary rehabilitation program starting 2 weeks after hospital discharge [34]. After the rehabilitation program patients significantly improved their lung function (vital capacity: + 11,3%) and exercise capacity (6 min walk distance: + 124 m) [34]. Therefore, it seems that pulmonary rehabilitation may play also an important part in the non-pharmacological treatment of post-COVID-19 patients with PICS and/or pulmonary fibrosis.

## Post-COVID phenotype “*the “black box”– or, the unmasking and/or exacerbation of underlying comorbidities*”

### Description

One other potential explanation of post-COVID sequelae is the unmasking, or exacerbation of underlying comorbidities [58, 59]. According to this concept, COVID-19 may exacerbate the health status of a population that is at high risk for developing chronic diseases. Also, pandemic restrictions may have interfered with regular health check-ups and preventive assessments which may have led to a deterioration of people’s health. Several large cohort studies concluded, that the risks of developing new diagnoses and symptoms involving the respiratory, cardiovascular, liver, kidney, or metabolic systems were several times higher in post-COVID patients compared to the non-COVID population [60–62].

### Potential rehabilitative interventions

It is often hard or almost impossible to differentiate if COVID-19 is the direct or indirect reason for the development of a new disease or symptom. If new diagnoses or symptoms can be objectively verified, established therapies should be applied. However, to further complicate matters, findings from a large population-based French cohort suggested that persistent symptoms in post COVID-19 patients may be associated more with the belief in having been infected with SARS-CoV-2, than with having a valid laboratory-confirmed SARS-CoV-2 infection [63]. Therefore, clinicians should carefully evaluate the health status of patients with persistent or new symptoms following COVID-19 to avoid, that symptoms due to another disease are erroneously attributed to post COVID-19 sequelae. This very special phenotype will always be a certain “black box” and represents a challenge for the management of COVID-19 patients. However, rehabilitative interventions should always be symptom-based.

## Unmet needs and future perspectives of rehabilitation in patients with persistent post-COVID symptoms

The WHO recommends, that hospitals and health care systems at regional, national, and global levels should prepare and be ready to hold sufficient clinical care capacities (staff, structure, supplies, and systems) to be able to provide appropriate care of all post COVID-19 patients [28]. This may include primary care providers (i.e. general practitioners), relevant specialists, outpatient rehabilitation programs, comprehensive inpatient/in-hospital multidisciplinary rehabilitation programs, mental health and psychosocial providers, or social care services. In addition, alternative delivery approaches such as home-based rehabilitation (with supervision via phone, video-call, or telemedicine), or the use of community outreach teams may also be useful [28]. Subsequently, various relevant aspects for the conduction of a successful post COVID-19 rehabilitation will be discussed.

### Maybe the most important question: is rehabilitation superior to natural recovery?

Existing evidence suggests that even after one year a relevant proportion of COVID-19 survivors still experience persistent symptoms involving various organ systems. It seems that female patients and those with more severe initial illness were more likely to suffer from COVID-19 sequelae after one year [64]. Importantly, large cohort studies have shown that there is a natural reconvalescence and a reduction of symptom prevalence within the first year after infection [65]. That raises the question whether rehabilitation might be superior to the effects of natural recovery. There is currently a large body of evidence suggesting that rehabilitation in post-COVID patients is beneficial. However, most of this evidence belongs to observational trials without control groups [66]. Up to now, there are only very few randomized, controlled trials available that investigated rehabilitative interventions like general exercise training (virtual or face-to-face) or respiratory muscle training programs in post-COVID patients [32, 67–71]. In summary, these studies found, that these kinds of rehabilitative interventions were superior to control groups to improve several important outcome measures like exercise performance, dyspnea, skeletal and respiratory muscle strength, or quality of life. However, randomized, controlled trials investigating the effects of a comprehensive and interdisciplinary rehabilitation program in people with post-COVID are still missing. Up to now, there are only data from an indirect comparison of two cohorts with comparable age and lung function available [72]. It was found, that at 6 weeks after hospital discharge, patients that underwent a comprehensive inpatient rehabilitation program showed a tremendously better physical performance compared to patients without a rehabilitative intervention (6-min walk distance: 380 m vs. 468 m) [72]. However, the benefits of comprehensive, multidisciplinary rehabilitation programs on COVID-19 symptoms, quality of life and working ability need to be investigated in future randomized, controlled trials.

### Identifying barriers and facilitators to rehabilitation

There are surely some barriers on the road to post COVID-19 rehabilitation. Although there is increasing evidence about the benefits of rehabilitation, there is still a huge lack of knowledge about the selection process of patients, that need rehabilitative interventions. Hence, also the awareness of the possibility of rehabilitation is scarce in patients but also often in physicians. It´s also sometimes difficult to determine which kind of rehabilitation service may be useful, especially when patients present a variety of symptoms across different organ systems (i.e. fatigue, dyspnea, pain, and cardiovascular symptoms). Finally, a barrier that belongs to each rehabilitation service: The application to request reimbursement for rehabilitation from health insurance is a bureaucratic and time-consuming procedure that might be avoided by patients and physicians.

However, there are also some facilitators to rehabilitation that are helpful. One of the positive side-effects of the pandemic was surely the exponential increase in the potential of telemedicine interventions. Tele-rehabilitation for example has been shown to be feasible and effective in post COVID-19 patients [69]. Also, the cooperation of multidisciplinary teams and the collaboration between different rehabilitation disciplines may enable optimal patient recovery [28].

### Identifying red flags for rehabilitation to deliver safe rehabilitation

The presence of existing red flags should be assessed before entering a rehabilitation program. Red flags are conditions that may cause an acute event or a deterioration of the current health status. To ensure safe rehabilitation, any significant cardiac impairment (i.e. myocarditis, pulmonary embolism, etc.) should be ruled out before starting an exercise training program [73]. Another aspect to provide safe rehabilitation refers to patients with a history of PEM. These patients should be closely supervised to detect thresholds that lead to symptom exacerbation. Consequently, the rehabilitation content needs to be adopted to avoid further flare-ups of symptoms. The application of pacing and energy conservation strategies is highly recommended, whereas intensive exercise training should not be applied [43].

### Setting realistic expectations

The aims and the patients´ expectations of the benefits of a rehabilitation program need to be realistic. Different from other rehabilitation conditions (i.e. learning to walk pain-free after a hip replacement surgery), patients with a post COVID-19 condition especially with fatigue are usually not completely cured after a rehabilitation program. Return to the pre-COVID health status takes time and the prognosis is unclear. Instead of only looking to a patient´s primary aim of reaching complete recovery, a relevant aim of rehabilitation should also rely on dealing with the current situation and supporting living with a (temporary) disability. Therefore, to avoid frustration, specific, but also realistic goal setting together with the health care professionals already at the beginning of a rehabilitation program is recommended [74].

### Providing responsible interaction

Post COVID-19 condition is probably the first disease that was created by patients. People affected by prolonged COVID sequelae exchanged their experiences on social media and created the term “Long COVID” [75]. Many people living with post COVID-19 sequelae often report that they have to face stigmatization and disbelief concerning their symptoms [75]. Rehabilitation staff should treat post COVID-19 patients respectfully and non-judgmental. Further, patients should not be blamed for symptom exacerbations (i.e. “your pacing was not very well”).

### Developing a new rehabilitation discipline `post COVID-19 rehabilitation

It is an international consensus, that rehabilitation programs should be individualized [31, 76]. People living with persistent post COVID-19 sequelae may present with a large heterogeneity of symptoms and impairments. A comprehensive assessment and anamnesis are necessary to identify treatable traits and to tailor an appropriate complexity of rehabilitative interventions [77]. However, further research is needed to identify optimal care pathway concepts.

**Fig. 2:**
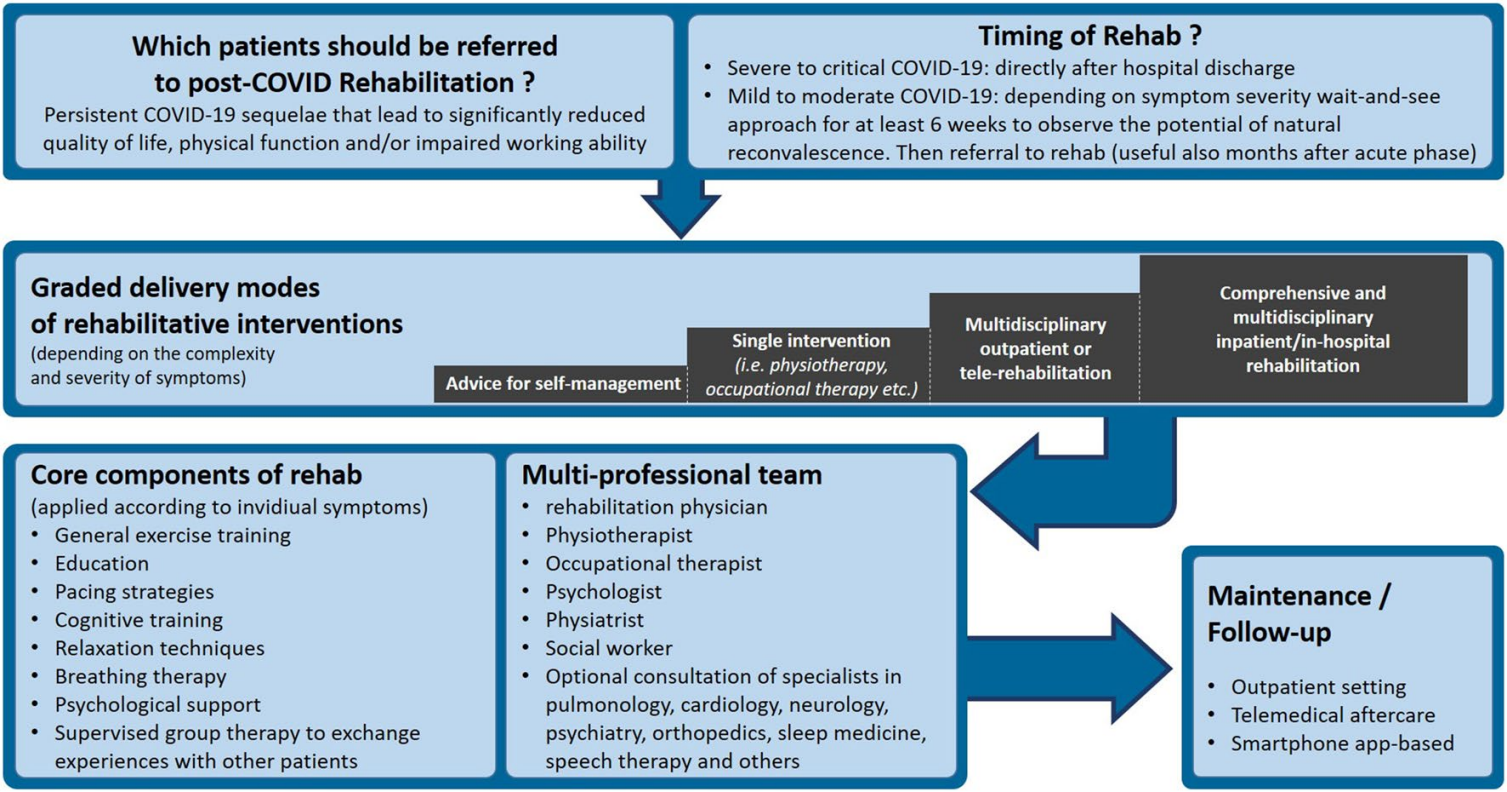
Proposal for post-COVID-specific rehabilitation concept

In rehabilitation medicine it is common, that rehabilitation programs are focusing on specific disciplines like orthopedics, neurology, cardiology, pulmonology, oncology, etc. With post COVID-19 condition the medical world is now facing the challenge that these patients often do not fit into one specific rehabilitation discipline because many patients present several burdensome symptoms that belong to various rehabilitation disciplines. Therefore, it seems crucial, that a post COVID-19-specific rehabilitation discipline needs to be created especially for patients with heterogeneous symptoms and significantly reduced quality of life, physical function, and/or impaired working ability [78]. Of course, it is also possible to adapt existing rehabilitation programs to the needs of post COVID-19 patients. There is a consensus in international rehabilitation guidelines that a comprehensive patient assessment and the need for individualized, multidisciplinary care are regarded useful [79]. Figure 2 provides a proposal concerning relevant aspects regarding the development of post COVID-19-specific rehabilitation programs. Further studies to evaluate COVID-19 specific rehabilitation programs and their contents are needed [80].

## Data Availability

Data sharing not applicable—no new data generated.
